# Investigating expectation and reward in human opioid addiction with [^11^C]raclopride PET

**DOI:** 10.1111/adb.12073

**Published:** 2013-07-05

**Authors:** Ben J Watson, Lindsay G Taylor, Alastair G Reid, Sue J Wilson, Paul R Stokes, David J Brooks, James F Myers, Federico E Turkheimer, David J Nutt, Anne R Lingford-Hughes

**Affiliations:** 1 Psychopharmacology Unit, University of BristolBristol, UK; 2 Oxford Health NHS Foundation TrustHigh Wycombe, UK; 3 Imperial College London, Centre for NeuropsychopharmacologyLondon, UK; 4 Department of Medicine, Division of Brain Sciences, Imperial College LondonLondon, UK; 5 Neuroimaging Department, Institute of Psychiatry, King's College LondonLondon, UK

**Keywords:** Addiction, dopamine, expectation, heroin, opioid, PET

## Abstract

The rewarding properties of some abused drugs are thought to reside in their ability to increase striatal dopamine levels. Similar increases have been shown in response to expectation of a positive drug effect. The actions of opioid drugs on striatal dopamine release are less well characterized. We examined whether heroin and the expectation of heroin reward increases striatal dopamine levels in human opioid addiction. Ten opioid-dependent participants maintained on either methadone or buprenorphine underwent [^11^C]raclopride positron emission tomography imaging. Opioid-dependent participants were scanned three times, receiving reward from 50-mg intravenous heroin (diamorphine; pharmaceutical heroin) during the first scan to generate expectation of the same reward at the second scan, during which they only received 0.1-mg intravenous heroin. There was no heroin injection during the third scan. Intravenous 50-mg heroin during the first scan induced pronounced effects leading to high levels of expectation at the second scan. There was no detectable increase in striatal dopamine levels to either heroin reward or expectation of reward. We believe this is the first human study to examine whether expectation of heroin reward increases striatal dopamine levels in opioid addiction. The absence of detectable increased dopamine levels to both the expectation and delivery of a heroin-related reward may have been due to the impact of substitute medication. It does however contrast with the changes seen in abstinent stimulant users, suggesting that striatal dopamine release alone may not play such a pivotal role in opioid-maintained individuals.

## Introduction

The euphoric effect of stimulants has been shown to correlate with striatal dopamine release, and increases in dopamine have also been reported with alcohol, nicotine and cannabis (Volkow *et al.* 2011), but not consistently nor always associated with a subjective ‘high’ (Boileau *et al.* 2003; Montgomery *et al.* 2007; Stokes *et al.* 2009). In contrast, we previously found that 50-mg intravenous heroin, despite producing a very profound ‘high’, did not increase dopamine levels in methadone-maintained opioid-dependent individuals (Daglish *et al.* 2008). However, there is evidence in primates that dopamine cell firing appears to be associated with the expectation, rather than the receipt of reward (see Schultz 2010). Consistent with this in humans, expectation of methylphenidate has been shown to result in larger increases in brain metabolism in cocaine-dependent individuals, than when it is unexpected (Volkow *et al.* 2003). Also dopamine release in response to cocaine cues has been demonstrated in the dorsal striatum of individuals abusing cocaine (Volkow *et al.* 2006; Wong *et al.* 2006). Increased dopamine levels in the right putamen have also been reported to heroin-related cues in abstinent opioid-dependent individuals (Zijlstra *et al.* 2008). Such increases in the dorsal striatum are consistent with its role in habit learning and compulsive drug use (Everitt & Robbins 2005). These increases in cue-induced dopamine levels occur despite chronic drug abuse being associated with a hypodopaminergic state (Volkow *et al.* 2010).

It is important to characterize the role of dopamine in the various drugs of abuse, because it has been argued that opioid and psychostimulant addiction are neurobiologically distinct with important implications for theories of addiction and its treatment (Badiani *et al.* 2011). We therefore developed our previous protocol using [^11^C]raclopride positron emission tomography (PET) imaging (Daglish *et al.* 2008), to further test two hypotheses in opioid-dependent participants: (1) striatal dopamine levels would increase with expectation of heroin reward and (2) striatal dopamine levels would increase after intravenous heroin.

## Materials and Methods

The study received approval from the Bath National Health Service Research Ethics Committee and the Administration of Radioactive Substances Advisory Committee, UK. All participants gave written informed consent prior to study procedures. Male participants aged over 18 years old, who met the Fourth Edition of the Diagnostic and Statistical Manual (American Psychiatric Association 1994) criteria for opioid dependence, currently maintained on methadone (≥30 mg/day) or buprenorphine (<8 mg/day), were recruited from outpatient addiction services. Participants were not dependent on or misusing any other substance other than nicotine, or had a history of clinically significant psychiatric or medical illness, or were taking other psychotropic medication. All participants provided a negative urinalysis for illicit substances of abuse before each scan.

### PET data acquisition

Each participant underwent three [^11^C]raclopride PET scans, separated by at least 1 week determined by scanner availability [mean ± standard deviation (SD) interval between first and second PET scans 14.0 ± 7.6 days; mean ± SD interval between second and third PET scans 12.5 ± 7.4 days]. Each PET scan occurred at a similar time of day and participants were over 24 hours after their last dose of methadone or buprenorphine, which they deferred taking until after the scan. At the first (‘reward’) PET scan, participants were told they would receive an injection of pharmaceutical heroin (diamorphine; subsequently referred to as heroin), although the dose was undisclosed as in our previous study (Daglish *et al.* 2008). Fifty milligrams of heroin in 5 ml of water was given intravenously, 55 minutes into the 100-minute scan. At their second (‘expectation’) PET scan, participants were also told that they would receive a heroin injection during the scan and again the dose was undisclosed. On this occasion, however, participants were given a low and undetectable dose of 0.1-mg heroin in 5 ml of water intravenously, 55 minutes into the 100-minute scan. In the reward and expectation scans we heightened their expectation of heroin reward by giving time checks every 10 minutes and counted down the last 30 seconds, and using a webcam so participants could see the injection, including the salient blood flashback. At their third (‘no-drug’) PET scan participants were told they would not receive an injection of heroin. This scan was used to measure [^11^C]raclopride binding, free from any expectation or rewarding effects. All participants underwent T1-weighted magnetic resonance imaging (MRI) on a separate day, with a Philips 1.5T Gyroscan Intera scanner (Philips, Best, The Netherlands) to produce structural images for reference.

All PET scans were conducted using an ECAT HR+ 962 scanner (CTI/Siemens, Knoxville, TN, USA), with an axial field of view of 15.5 cm. A 10-minute transmission scan was acquired prior to each emission scan to correct for tissue attenuation. Dynamic emission scans were acquired in three-dimensional mode using a standard acquisition protocol (38 frames over 100 minutes; Stokes *et al.* 2010). Each participant received 240 MBq of [^11^C]raclopride administered as an initial intravenous bolus (922 ml/hour for 60 seconds) followed by a constant infusion (8.8 ml/hour) for the remainder of the scan (Watabe *et al.* 2000). We used a bolus infusion of [^11^C]raclopride to deal with potential limitations of our previous bolus protocol such as timing of heroin and [^11^C]raclopride injection and minimize confounds such as drug-related blood flow effects by establishing a state of constant equilibrium (Carson *et al.* 1997).

### Subjective and physiological measures

Before each [^11^C]raclopride PET scan, participants completed Beck's Depressive Inventory (BDI; Beck *et al.* 1961) and Spielberger State-Trait Anxiety Inventory (SSAI, STAI; Spielberger *et al.* 1983). Visual analogue scales (VAS; 100 points) were used to rate ‘high’, ‘rush’, ‘gouched’ (local slang for opioid intoxication), ‘urge and craving’ (to use heroin) and ‘withdrawal’ during their [^11^C]raclopride PET scans. Ratings were collected at baseline, 6 minutes prior to the start of the scan, and at 6, 16, 28, 38, 48, 63, 73, 83, 93 and 5 minutes post-scan. Opioid withdrawal was also assessed before and after each scan using a Modified Himmelsbach Opiate Withdrawal Scale (Law *et al.* 1997). Levels of expectation were measured using VAS, asking participants how good they expected the scan and heroin injection to be immediately before the scan and how good it had been immediately after the scan. They were also asked what equivalent value of street heroin they had been given after reward and expectation scans.

To objectively assess the effects of heroin on the brain we used our standard protocol measuring saccadic eye movements immediately before each VAS data collection point (Wilson *et al.* 1993). The impact of heroin on respiratory depression was monitored with pulse oximetry. Blood for methadone plasma concentrations was taken immediately before each scan and quantified using a modified liquid–liquid extraction followed by gas chromatography–mass spectrometry (Paterson, Cordero & Burlinson 2004).

### PET data analysis

All dynamic scans were motion corrected using frame-by-frame realignment in Piwave 7.0 (in-house software; Imperial College London, London, UK) running in Matlab® (The MathWorks Inc., Natick, MA, USA; Montgomery *et al.* 2006). The resultant dynamic frames were processed with receptor parametric mapping (RPM) to generate an add-image (Gunn *et al.* 1997), which was a weighted sum of all 38 frames. MRIs were co-registered to the individual add-images using Statistical Parametric Mapping 5 (SPM5; Functional Imaging Laboratory, University College London, UK) and then normalized into Montreal Neurological Institute (MNI) space (International Consortium for Brain Mapping) using bias-corrected segmentation in SPM5.

For the region of interest (ROI) analysis, the inverse deformation parameters obtained from the segmentation process were applied to a functional striatal atlas (Martinez *et al.* 2003). Striatal and cerebellar ROIs were defined using an atlas composed of the three functional subdivisions of the striatum: limbic (ventral), associative (pre-commissural dorsal putamen, pre-commissural dorsal caudate and post-commissural dorsal caudate) and sensorimotor striatum (post-commissural putamen) and cerebellum. The individual deformed atlases were used to sample the add-images described earlier using Analyze software (http://www.analyzedirect.com;). Accounting for radioactive decay, we previously reported [^11^C]raclopride equilibrium stability of 0.06% per minute over the equilibrium period using this bolus infusion protocol (Stokes *et al.* 2009).

**Figure 1:**
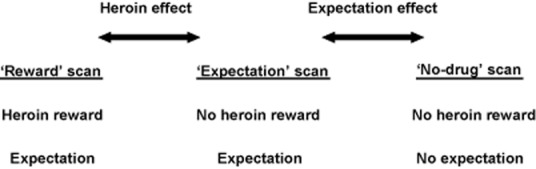
Drug and expectation factors present in each participants' scan

Binding potential (BP_ND_) values for [^11^C]raclopride in each striatal region were calculated as the ratio of striatal counts to cerebellar counts, minus 1, over the steady-state time periods (Lammertsma & Hume 1996). Frames 19–22 (35.5–55.5 minutes) corresponded to the pre-heroin injection equilibrium period and were used to generate ‘early’ steady-state phase add-images. Frames 25–38 (57.5–100.5 minutes) corresponded to the post-heroin injection equilibrium period and were used to generate ‘late’ steady-state phase add-images (Egerton *et al.* 2009). The study was designed so that any difference in [^11^C]raclopride binding between reward and expectation scans would be due to the effect of heroin, while differences in [^11^C]raclopride binding between expectation and no-drug scans would be due to the effect of expectation (see Fig. 1).

Exploratory voxelwise analysis was also undertaken using image algebra within SPM5 running in Matlab® (The MathWorks Inc.) to create parametric BP_ND_ maps. The steady-state add-image voxels were divided by the cerebellar counts minus 1. Percentage increase images between the early and late phase BP_ND_ maps were calculated for reward, expectation and no-drug scans using the equation 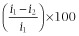, where *i*_1_ is the early phase parametric image and *i*_2_ is the late phase parametric image. The resulting images were normalized to a [^11^C]raclopride template image in MNI space and smoothed using a 6 mm^3^ full width at half-maximum Gaussian kernel.

### Statistical analysis

All statistical comparisons, apart from voxelwise analyses, were performed using SPSS 18.0 (SPSS, Chicago, IL, USA). All *t*-tests were two-tailed. Paired samples *t*-tests were used to compare subjective measures. Correlations between parametric data were assessed using Pearson's product–moment correlation coefficient. Differences in early and late steady-state phases within and between different scans were investigated using repeated measures analysis of variance (ANOVA), with striatal region as a within-subject factor. Heterogeneity of covariance was tested with the Mauchly sphericity test and degrees of freedom modified using the Greenhouse–Geisser adjustment, where appropriate.

Voxelwise analyses were performed within SPM5 using an explicit striatal mask. Differences in early and late steady-state phases within and between different scans were compared using a paired *t*-test. A height threshold of *P* < 0.001 and volume-corrected cluster threshold of *P* < 0.05 were used for statistical significance (Stokes *et al.* 2009).

## Results

Eleven opioid-dependent individuals started the three-scan protocol; however, one withdrew after the first scan due to subsequent failure of [^11^C]raclopride synthesis, leaving 10 who completed all three [^11^C]raclopride PET scans. The mean ± SD participant age was 37.8 ± 9.0 years. Nine participants were receiving methadone maintenance (mean ± SD daily dose 50.5 ± 25.2 mg) and one buprenorphine maintenance (5 mg/day). The mean ± SD length of prior opioid use was 13.9 ± 6.3 years. All participants were tobacco smokers and had last smoked cannabis more than 2 months prior to imaging. Six participants had no recent alcohol consumption, with the remaining participants consuming 8–18 units (64–144 g) of alcohol per week. Occasional stimulant use was reported by all participants, with last use over 2 years previously for all but two (2 months, 6 months). Mean ± SD BDI and SSAI scores did not differ significantly between scans and were as follows: reward scan (BDI 12.9 ± 9.5; SSAI 39.1 ± 12.8), expectation scan (BDI 12.6 ± 10.1; SSAI 34.5 ± 8.8) and no-drug scan (BDI 12.9 ± 10.9; SSAI 33.3 ± 9.7). The mean ± SD STAI score was 43.7 ± 11.3. These scores reflect only mild symptoms of anxiety and depression.

### Subjective and physiological measures

**Figure 2:**
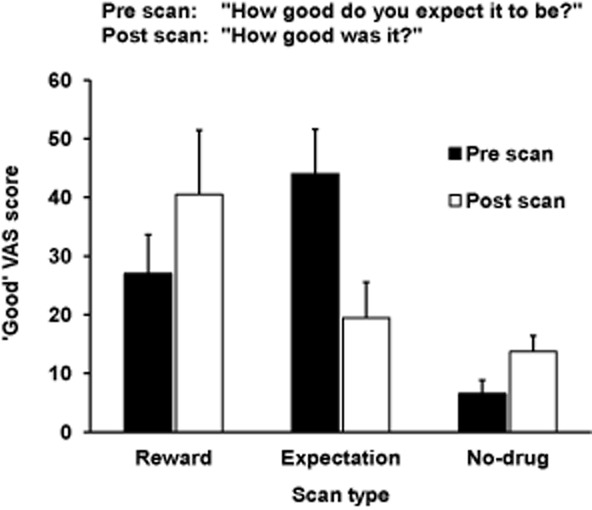
Mean ‘good’ visual analogue scale (VAS) scores, pre and post each scan. Standard error bars shown

The key experimental objective of inducing expectation for heroin reward in both reward and expectation scans was achieved (Fig. 2). Expectation levels as measured by mean pre-scan ‘good’ VAS scores were higher for the expectation scan than the reward scan (*t*_9_ = −2.18, *P* = 0.057). A significant difference was seen between mean pre-scan ‘good’ VAS scores of reward and no-drug scans (*t*_9_ = 3.11, *P* = 0.013), and of expectation and no-drug scans (*t*_9_ = 5.61, *P* < 0.001). All participants in the reward scan and 7 of 10 participants in the expectation scan believed they had experienced effects of heroin after the injection. The mean ± SD estimated equivalent value of street heroin received was £18.00 ± 13.20 in the reward scan and £5.50 ± 4.40 in the expectation scan (£10 buys approximately 0.2-g heroin).

**Figure 3:**
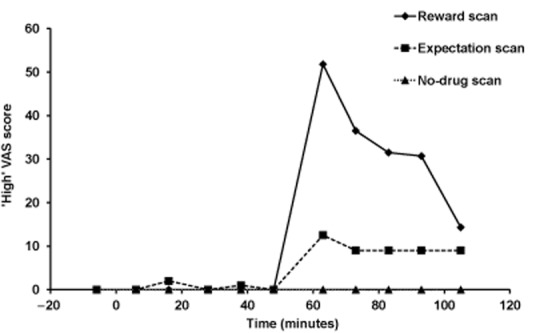
Mean ‘high’ visual analogue scale (VAS) scores, plotted against time for each scan

In the reward scan following heroin injection, there were pronounced and significant increases in VAS scores for the positive effects of heroin (Fig. 3) such as high (mean ± SD change 51.8 ± 30.6, *t*_9_ = 5.36, *P* < 0.001), rush (47.8 ± 37.7, *t*_9_ = 4.01, *P* = 0.003) and gouched (29.0 ± 31.8, *t*_9_ = 2.89, *P* = 0.018). There were smaller and non-significant increases seen in these VAS scores in the expectation scan and in the no-drug scan, all participants scored zero throughout. During all scans ratings of withdrawal and urge and craving to use heroin were low and did not change significantly as a result of any interventions.

The predicted slowing and impairment of saccadic eye movements was observed in the reward scan, but not in the expectation scan consistent with our previous study (Daglish *et al.* 2008). Oxygen saturation levels remained stable and within normal limits in all participants throughout the expectation and no-drug scans, but fell in the reward scan to a mean ± SD minimum level of 83.3% ± 10.3.There was no significant difference seen in pre-scan methadone plasma levels between the three scans (mean ± SD reward scan 255.6 ± 201.4 ng/ml; expectation scan 228.9 ± 229.4 ng/ml; no-drug scan 210.0 ± 160.4 ng/ml; ANOVA, *F*_2,14_ = 0.37, *P* = 0.70).

### [^11^C]raclopride PET

No significant effect of expectation on [^11^C]raclopride BP_ND_ in any striatal ROI was found in two comparisons: [^11^C]raclopride BP_ND_ in early and late steady-state phases within the expectation scan (ANOVA *F*_1,9_ = 0.08, *P* = 0.78), and between early and late steady-state phases in the expectation and no-drug scans (ANOVA *F*_1,9_ = 0.32, *P* = 0.59). We also found no significant effect from 50 mg of heroin on [^11^C]raclopride BP_ND_ values in any striatal ROI. Two comparisons were undertaken: [^11^C]raclopride BP_ND_ in early and late steady-state phases within the reward scan (ANOVA *F*_1,9_ = 0.06, *P* = 0.82), and between early and late steady-state phases in the reward and expectation scans (ANOVA *F*_1,9_ = 0.11, *P* = 0.75). In addition there were no significant correlations between VAS high scores and [^11^C]raclopride BP_ND_ values in any striatal region. Table 1 shows mean BP_ND_ values in each of the scans.

**Table 1:** Mean ± standard deviation [^11^C]raclopride binding potential in each scan

Striatal region	Reward scan		Expectation scan		No-drug scan	
	Early	Late	Early	Late	Early	Late
Sensorimotor						
Left	2.44 ± 0.33	2.45 ± 0.28	2.47 ± 0.26	2.48 ± 0.27	2.57 ± 0.31	2.54 ± 0.36
Right	2.32 ± 0.38	2.34 ± 0.33	2.44 ± 0.30	2.44 ± 0.30	2.58 ± 0.36	2.56 ± 0.39
Associative						
Left	1.97 ± 0.33	1.96 ± 0.29	2.02 ± 0.30	1.99 ± 0.27	2.19 ± 0.32	2.11 ± 0.33
Right	2.19 ± 0.31	2.21 ± 0.29	2.25 ± 0.29	2.22 ± 0.28	2.37 ± 0.33	2.31 ± 0.35
Limbic						
Left	1.95 ± 0.31	1.96 ± 0.26	2.10 ± 0.20	2.11 ± 0.20	2.26 ± 0.37	2.18 ± 0.36
Right	1.91 ± 0.29	1.90 ± 0.22	1.90 ± 0.26	1.90 ± 0.28	1.99 ± 0.32	2.01 ± 0.25

Early = early steady-state phase; Late = late steady-state phase.

Our exploratory voxelwise analysis generally confirmed the ROI analysis with no significant differences found in early and late steady-state phases between reward and expectation scans or between expectation and no-drug scans. There were small clusters in the reward and expectation scans in which BP_ND_ increased significantly from early to late steady-state phases (Table 2); no significant decreases were seen. There were no significant increases or decreases in BP_ND_ within the no-drug scan.

**Table 2:** Areas of significant increase in [^11^C]raclopride binding potential from early to late steady-state phases within reward and expectation scans, identified in the exploratory voxelwise analysis

Striatal region	Cluster size^a^ (voxels)	Coordinates^c^ (x, y, z)	t value	Corrected^b^ P value
Reward scan				
L associative/limbic	43	−12, 22, −6	9.42	< 0.001
L limbic	15	−24, 8, −12	6.89	0.008
Expectation scan				
R sensorimotor	38	28, −4, −8	12.02	< 0.001
L limbic	35	−24, 2, −12	7.3	< 0.001
L limbic	26	−18, 20, −8	8.25	0.001
R associative	13	18, 20, 8	7.51	0.021

a Cluster sizes correspond to number of voxels of dimension 2 mm^3^.

b *P* values were after correction for striatal volume.

c The Montreal Neurological Institute coordinates, corrected *P* and *t* values are for the voxel in each cluster containing the peak effect size. L = left; R = right.

## Discussion

This is the first study to our knowledge to examine whether the expectation of heroin reward increases striatal dopamine levels in human opioid addiction. Despite our experimental paradigm producing marked expectation, no increase in dopamine levels in any striatal region was detected. In addition there was no increase in regional striatal dopamine levels following a rewarding dose (50 mg) of intravenous heroin.

Salient drug cues and giving heroin in their first [^11^C]raclopride PET scan resulted in increased levels of expectation at the second scan in all participants. We enhanced the cues for the opioid-dependent participants compared with our previous study (Daglish *et al.* 2008). Because passive administration of drugs has been shown to result in smaller increases in dopamine compared with a self-administration (Leyton 2007), a webcam was used so the participants could see the highly salient ‘flashback’ and injection of heroin. Leyton emphasizes the importance of cues in his ‘two-factor dopamine model’ based on evidence from studies with psychostimulants (Leyton 2007). However, his model posits that low dopamine responsivity would be associated with the absence of cues rather than the salient cues in our study. Despite our highly salient injection procedure, we were still not able to show any changes in striatal dopamine levels in substitute-maintained opioid-dependent participants.

Previous neuroimaging studies in drug addiction have reported increased dopamine levels in abstinent individuals associated with salient videos, but have measured ‘craving or urge’, ‘intentions to use’, ‘desire’ or ‘need or want’ rather than ‘expectation’ (Volkow *et al.* 2006, 2010; Wong *et al.* 2006; Zijlstra *et al.* 2008). While there are similarities between expectation and craving, expectation generally implies looking forward to something positive or good, whereas craving has a sense of need and urgency that may be more present in abstinence. In contrast to expectation, craving to use did not vary during the scans, probably because participants knew they were going to receive heroin and/or their substitute medication. It is therefore possible that increased striatal dopamine levels are associated with craving rather than expectation.

Expectation of therapeutic benefit from a dopaminergic agonist in patients with Parkinson's disease has been shown to increase dopamine levels in the ventral striatum and perception of clinical benefit to increases in the dorsal striatum (de la Fuente-Fernández *et al*. 2002). Similarly expectation of analgesia has been shown to be associated with increased dopamine levels in the striatum (Scott *et al.* 2007). The anticipation of therapeutic benefit in patients with Parkinson's disease has been likened to the expectation of reward. However, patients expect to gain relief from their physical disorder, which is akin to negative rather than positive reinforcement. Consequently increased dopamine may be associated with relief from a negative state (cf. bupropion in smoking cessation), whereas in our study, the opioid-dependent participants were not in withdrawal and instead experienced a ‘high’ from their substance of abuse. Another factor may be that there was 100% certainty they would receive heroin because a study with Parkinson's patients reported that striatal dopamine levels increased maximally with 75% certainty and not at all with 100% certainty (Lidstone *et al.* 2010). Our results are however consistent with other studies where expectation of caffeine in habitual coffee drinkers or of intravenous glucose in healthy volunteers was not associated with increased striatal dopamine levels (Kaasinen *et al.* 2004; Haltia *et al.* 2008).

Our findings also speak to Schultz's work in primates implicating dopamine neuronal firing in learning and expectation of reward, on which we based our hypothesis (Schultz 2010). Schultz, Dayan & Montague (1997) showed that after repeated pairings of cues followed by reward, the timing of dopamine neuron activation changed from just after reward delivery to the time of cue onset, with a loss of dopaminergic response to the predicted reward itself. Because reward was defined as involving a variety of processes including hedonic feelings and generating approach behaviour, it may be that dopamine is more associated with the latter, which did not occur in our study because they were given the drug. However, Schultz also showed that if the conditioned cue was presented and no subsequent reward delivered, a reduction in dopamine neuron activity was seen. Consistent with this, our voxelwise analysis of the expectation scan showed small clusters of reduced dopamine levels after the non-rewarding dose (0.1 mg) of heroin. While we report this finding cautiously because it was found on exploratory analysis, it is to our knowledge the first evidence in humans of dopamine's involvement in reward prediction error.

We again found that a rewarding dose (50 mg) of heroin did not result in significant increases in striatal dopamine in opioid-maintained individuals (see also Daglish *et al.* 2008). Our exploratory voxelwise analysis revealed small clusters of reduced dopamine levels that are consistent with a study of another opioid agonist, alfentanil, in healthy volunteers (Hagelberg *et al.* 2002). In contrast a remifentanil infusion has been reported to increase striatal dopamine levels measured with [^18^F]fallypride PET in healthy volunteers and abstinent alcohol-dependent individuals, but there was no relationship to craving (Spreckelmeyer *et al.* 2011).

Our finding that striatal dopamine levels were not increased by a rewarding dose of heroin is consistent with studies in cocaine or alcohol addiction, showing blunted methylphenidate-induced or amphetamine-induced dopamine release (Volkow *et al.* 1997; Martinez *et al.* 2005, 2007). Methylphenidate-stimulated dopamine release has also been shown to be blunted in abstinent opioid-dependent individuals although no subjective effects were reported (Martinez *et al.* 2012). However, importantly, neither amphetamine nor methylphenidate are the participants' ‘drug of choice’ given by the usual route, as was the case in our study. It appears therefore that changes in dopamine can occur in the absence of pronounced subjective effects, and that the rewarding effects of opioids can occur in the absence of detectable dopamine levels.

While the impact of drug-taking status and opioid maintenance medications on the dopaminergic system remains to be fully characterized, it may have been an important factor in our experiments. Cue-induced increases in dopamine have been reported in abstinent cocaine-dependent or opioid-dependent individuals (Volkow *et al.* 2006; Wong *et al.* 2006; Zijlstra *et al.* 2008), whereas the opioid-dependent participants in both of our studies were maintained on methadone (except one participant on buprenorphine) and injecting heroin intermittently (note it would be unethical and dangerous to administer heroin to non-maintained individuals). Pre-clinical evidence suggests that acute administration of methadone blocks dopamine receptors and increases dopaminergic turnover, whereas in methadone addicts lower levels of cerebrospinal fluid homovanillic acid suggest reduced dopamine turnover (Tamarkin, Goodwin & Axelrod 1970; Sasame *et al.* 1972; Clemens & Sawyer 1974). Therefore a reduction in dopamine turnover could result in difficulty in detecting any dopamine increase with ‘expectation’ or ‘high’, which may have been the case in the present study. It may also be that part of methadone's efficacy as a maintenance treatment is due to its ability to blunt dopaminergic responses, thereby reducing craving and cue reactivity to heroin.

Both our studies, showing no changes in striatal dopamine in opioid-maintained individuals, are however consistent with evidence from animal models. Unlike for cocaine, studies have shown that opioid reinforcement is not critically dependent on an intact mesolimbic dopaminergic system (Pettit *et al.* 1984; Van Ree & Ramsey 1987; Gerrits & Van Ree 1996). High impulsivity in rats is associated with low D_2/3_ receptor levels in the ventral striatum, and increased vulnerability to lose control over cocaine intake and relapse to cocaine seeking after abstinence but not to heroin use (Dalley *et al.* 2007; McNamara *et al.* 2010). Martinez *et al*. (2012) also reported that unlike their studies with cocaine-dependent individuals, methylphenidate-stimulated dopamine release did not correlate with heroin self-administration. A recent comparison also strongly argued for important distinctions between psychostimulant and opioid addiction, including in the dopamine system (Badiani *et al.* 2011). We therefore suggest that there is good evidence for dopamine playing a different role depending on the substance of abuse.

While PET has been a highly useful tool for studying neurotransmitter systems in man, its sensitivity is limited. It is therefore possible that any dopamine release induced either by the expectation of heroin or heroin itself is too small or localized to be detected by current [^11^C]raclopride PET scan and analysis techniques. It is also possible that the sample size of 10 participants was too small to detect any dopamine release. The study was adequately powered (0.8) to detect a 3.6% change in overall [^11^C]raclopride binding consistent with other studies that reported significant changes of 4–5% in [^11^C]raclopride binding with cue exposure (Volkow *et al.* 2006; Wong *et al.* 2006). The power calculation used a within-volunteer SD of 3.6% for overall striatal [^11^C]raclopride binding, calculated from our previously reported test–retest study (Stokes *et al.* 2010). Significant increases in dopamine to treatment expectation (22%; de la Fuente-Fernández *et al*. 2002), stimulant administration (16%; Volkow *et al.* 2002) and alcohol administration (15%; Boileau *et al.* 2003) have also been reported with similar or lower participant numbers. In addition, we saw both increased and decreased BP_ND_ values in all striatal areas without a consistent trend. Using the observed differences between ‘early versus late’ phases and between scans, over a hundred participants would have had to be scanned to generate a significant finding that is clearly impracticable and unethical. The study had to overcome two major challenges: firstly that participants were required to attend for four scans (three PET and one MRI), and secondly the recruitment of opioid-dependent individuals without co-morbid stimulant or benzodiazepine abuse. Consequently, to ensure a sufficient number of such participants were recruited to robustly test our hypotheses, the study took 5 years to complete. A further limitation of all [^11^C]raclopride PET studies is the uncertainty about what any differences in BP_ND_ represent. While changes are measurable following both pharmacological and psychological interventions, these may reflect either changes in synaptic dopamine concentration or in dopamine D_2/3_ receptor availability (Laruelle 2000; Egerton *et al.* 2009).

In conclusion, we have demonstrated that striatal dopamine release does not appear to play a key role in mediating either the expectation or ‘high’ associated with heroin use in opioid-dependent individuals maintained on substitute medication. Given how many opioid-dependent individuals are in maintenance programmes, it is vital that we understand the impact of substitute medications on the dopaminergic system. In addition, there is growing evidence that dopamine is likely to play differing roles in opioid addiction compared with alcohol and cocaine addiction (Badiani *et al.* 2011). Our results provide further evidence for the debate about the role of dopamine in opioid addiction because studies in abstinence and those on substitute medication suggest its role may differ, and the impact on clinical outcomes deserves further investigation.
